# Influence of Hydrogen Peroxide on Disinfection and Soil Removal during Low-Temperature Household Laundry

**DOI:** 10.3390/molecules27010195

**Published:** 2021-12-29

**Authors:** Petra Forte Tavčer, Katja Brenčič, Rok Fink, Brigita Tomšič

**Affiliations:** 1 Faculty of Natural Sciences and Engineering, University of Ljubljana, Aškerčeva 12, 1000 Ljubljana, Slovenia; katja.brencic@ntf.uni-lj.si (K.B.); brigita.tomsic@ntf.uni-lj.si (B.T.); 2 Faculty of Health Sciences, University of Ljubljana, Zdravstvena pot 5, 1000 Ljubljana, Slovenia; rok.fink@zf.uni-lj.si

**Keywords:** textile care, low-temperature washing, laundry hygiene, hydrogen peroxide, disinfection activity, soil removal, colour difference, tensile properties

## Abstract

In the Water, Energy and Waste Directive, the European Commission provides for the use of household washing programmes with lower temperatures (30–40 °C) and lower water consumption. However, low washing temperatures and the absence of oxidising agents in the liquid detergents, and their reduced content in powder detergents, allow biofilm formation in washing machines and the development of an unpleasant odour, while the washed laundry can become a carrier of pathogenic bacteria, posing a risk to human health. The aim of the study was to determine whether the addition of hydrogen peroxide (HP) to liquid detergents in low-temperature household washing allows disinfection of the laundry without affecting the properties of the washed textiles even after several consecutive washes. Fabrics of different colours and of different raw material compositions were repeatedly washed in a household washing machine using a liquid detergent with the addition of 3% stabilised HP solution in the main wash, prewash or rinse. The results of the antimicrobial activity, soil removal activity, colour change and tensile strength confirmed the excellent disinfection activity of the 3% HP, but only if added in the main wash. Its presence did not discolour nor affect the tensile strength of the laundry, thus maintaining its overall appearance.

## 1. Introduction

Among the measures to reduce greenhouse gas emissions from households and to reduce energy consumption, the European Commission has mandated in the Water, Energy and Waste Directive that household washing programs with lower temperatures (30–40 °C) and lower water consumption must be used [1]. Given the huge amounts of laundry we wash in households today (clothing consumption is constantly increasing due to the rise of fast fashion), such an approach makes sense and can actually bring measurable savings in energy and water consumption [2,3]. LCA studies of clothing, detergents and washing machines show that the use phase is generally the most energy consuming phase in the life cycle of clothing, ahead of the production and transport phases [4]. Consumer washing habits vary [5,6], but in the last 20 years the preferred washing temperatures worldwide have been between 30 °C and 60 °C [4,5,6,7,8]. However, these washing methods cannot guarantee the sufficient cleanliness of the laundry and especially the sufficient disinfection of the laundry [9]. It is worth noting that any pathogen associated with human disease is likely to be found on textile clothing [10]. Since these are environmental pathogens, contamination of textile surfaces could have occurred after or during the laundering process. Accordingly, inadequate domestic laundry hygiene can be of great concern to certain at-risk groups (e.g., immunocompromised individuals, the elderly, young children, etc.), as contamination or recontamination from hygienically inadequately cleaned textiles or even cross-contamination between household members could cause certain health problems [11,12,13,14]. It has been found that hygienic textiles can only be achieved at a washing temperature above 60 °C [15]. Accordingly, it is realistic to assume that potentially pathogenic microorganisms are not eliminated when infected laundry is washed at temperatures of 30–40 °C, especially since these temperatures are ideal for their development and multiplication [7,11,16].

The problem of textile washing hygiene is partly solved by oxidizing agents added to detergents in the form of perborates or percarbonates (bleach activators) [9,11,17]. Under suitable conditions, active oxygen is released into the wash bath where it oxidises coloured stains while acting against a variety of microorganisms (bacteria, bacterial spores, fungi, yeasts and viruses) [18,19,20]. Bleach activators are found only in solid detergent powders, but their share has been decreasing since 2010 due to EU environmental regulations. Liquid detergents, which are popular among consumers, do not contain bleach activators at all because they are unstable in liquid formulations [21]. Therefore, the result of using liquid detergents with a low-temperature laundry care program manifests itself in hygienically unsafe textiles and internal parts of the washing machine, which offers the possibility of spreading potentially pathogenic microorganisms through textile products [15,22,23]. Achieving adequate hygiene of laundry and internal parts of the washing machine during low-temperature washing is a particular problem with regard to current and future epidemics [24].

In order to achieve a balance between energy saving, adequate hygiene and effective soil removal, it is crucial to optimise the process of household laundry by introducing cold disinfection. Accordingly, the use of ecologically acceptable agents that do not pollute wastewater, are safe for humans and do not pose a risk for the development of long-term resistance of microorganisms due to increased use is urgently needed.

Among antimicrobial agents, hydrogen peroxide (HP) and peroxyacetic acid (PAA) are among the most environmentally friendly disinfectants [25,26]. They are characterised by high efficiency, broad spectrum antimicrobial activity, low toxicity and ease of use. HP is an antiseptic and antibacterial agent against various gram-negative and positive bacteria. Since HP efficacy is concentration-dependent, the presence of catalase and peroxidases in the cell can substantially decrease the antibacterial effect, and therefore concentrations below 3% are less effective [27]. Studies in the field of ensuring effective disinfection of laundry during the washing process with the addition of HP are limited in number and mostly oriented in the field of professional textile care [28]. Accordingly, the disinfection activity of the HP/PAA mixture of hospital laundry when washed at 40 °C was well studied [29]. Washing at 40 °C was found to reduce energy consumption while showing adequate disinfection activity against selected microorganisms, *Enterococcus faecium*, *Staphylococcus aureus*, *Enterobacter aerogenes* and *Candida albicans*, but at a higher concentration of HP/PAA. Compared to washing at 60 °C, washing at 40 °C showed the least mechanical damage of the standard cotton control. The wastewater showed some degree of soiling and was biodegradable, thus suitable for biological treatment. The antibacterial activity of HP during cold washing at 30 °C was also investigated using *Enterococcus faecium* and *Enterobacter aerogenes* as model thermoresistant Gram-positive and Gram-negative bacteria. The result showed excellent growth reduction of both tested bacteria, but the latter was time-dependent. Accordingly, the highest antibacterial activity was obtained during the main wash with a duration of 43 min [28]. In addition to bactericidal and fungicidal activity, the efficacy of HP against *Clostridiodes dificile* spores in the industrial tunnel washing of hospital laundry was also demonstrated, showing their sporicidal activity [30].

To the best of our knowledge, there is no study on the overall effect of HP addition in low-temperature household laundry (30–40 °C), which would provide systematic data on the laundry hygiene and stain removal efficiency, and its influence on colour changes and mechanical properties of textiles. Therefore, despite the extreme importance of providing hygienically adequate textile surfaces during household washing, which is one of the effective means of controlling the spread of pathogenic microorganisms and the occurrence of diseases, the introduction of HP in the household washing process remains an important, complex and largely unexplored research task. Accordingly, the aim of this study was to determine whether the addition of HP in the form of a 3% solution to a liquid detergent provides efficient disinfection and effective textile care during the household washing at 40 °C, and whether the use of 3% HP affects the properties of textiles. Moreover, the influence of the wash cycle on the overall effect of the 3% HP addition was also monitored. For this purpose, 3% HP was added during the main wash cycle, the prewash or during the rinse cycle. For each wash cycle, the disinfection activity was determined. The same washing procedures were used to test the removal of four different EMPA standard soils to give an insight on the stain removal efficiency. Influence on colour differences and tensile strength of the laundry were studied after consecutive washings of the randomly selected textile samples of different composition and colour with and without the addition of 3% HP in each wash cycle.

## 2. Results and Discussion

### 2.1. Disinfection Activity of 3% HP

**Figure 1:**
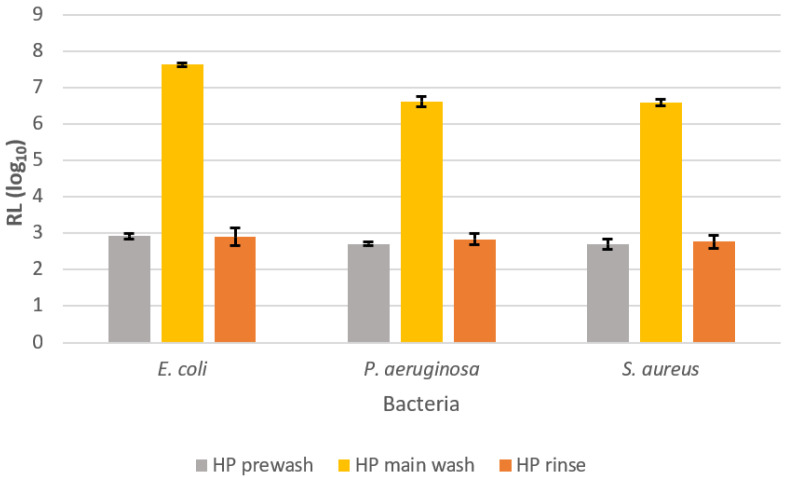
Disinfection activity, RL (log CFU cm^−2^), of 3% hydrogen peroxide added in the prewash, main wash and rinse cycles against tested bacteria *E. coli*, *P. aeruginosa* and *S. aureus*.

**Figure 2:**
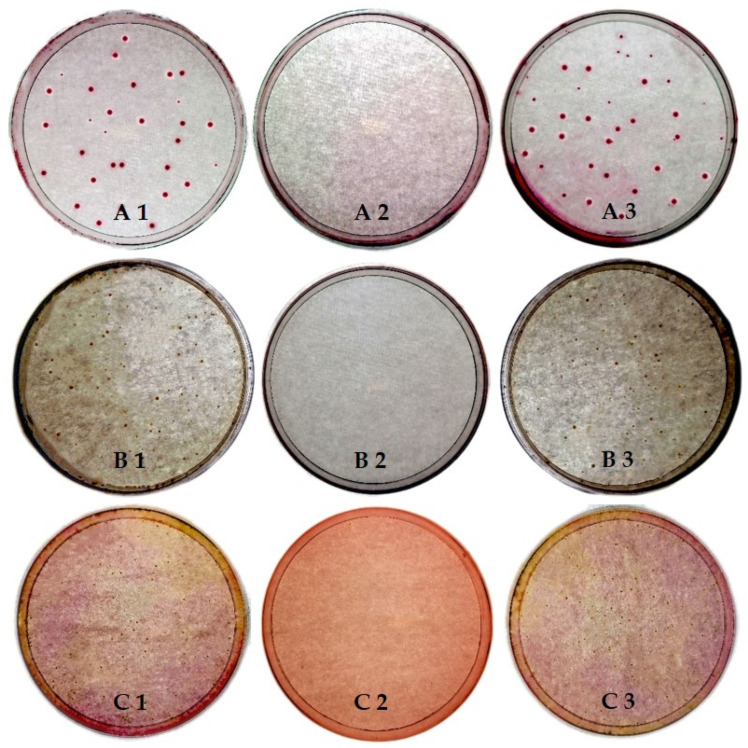
Agar plates of *E. coli* (**A**), *P. aeruginosa* (**B**) and *S. aureus* (**C**) growth after studied wash cycles in the presence of 3% HP as disinfectant agent: (**1**) prewash, (**2**) main wash and (**3**) rinse.

Prior to testing the influence of the HP addition in the washing process on the appearance and performance of textile goods, its disinfection activity was investigated. The focus was on determining the most appropriate wash cycle for the HP addition that would reflect the highest disinfection efficiency. Accordingly, 3% HP was added during the wash, i.e., prewash, main wash and rinse. The results are presented in Figure 1 and Figure 2. As can be seen in Figure 1, the addition of 3% HP caused some disinfection activity when added at the beginning (prewash) or at the end (rinse) of the wash, resulting in a 2.7–2.9 log reduction in the growth of the tested bacteria. On the other hand, the addition of 3% HP in the main wash cycle showed excellent inactivation of the tested bacteria, corresponding to more than 99.9999% reduction in bacterial growth. Accordingly, the highest disinfection activity was obtained against *E. coli* with a growth reduction of 7.62 log, followed by *P. aeruginosa* with a reduction of 6.61 log and *S. aureus* with a reduction of 6.58 log. The extreme increase in disinfection activity of 3% HP in the main wash compared to the prewash and rinse can be explained by the longer contact time of 3% HP with the tested bacteria, as well as higher temperature. Namely, in comparison to the prewash and rinse, the duration of the main wash was about 2.5 and 5 times longer, with 12–14 °C higher temperature of the washing bath. Accordingly, the prolonged duration of the main wash allowed a longer contact time of the tested bacterial cells with the extremely biocidal hydroxyl radicals (OH^•^) formed during the activation of HP in the presence of trace amounts of transition metal ions (known as the Fenton reaction) present in tap water, while higher temperature conditioned the faster activation reaction of HP [31,32]. It is assumed that generated OH^•^ reacts with bacterial cell lipids, proteins and nucleic acids to result in the break of RNA and DNA and the destruction of sulfhydryl bonds in proteins and membranes [33]. Since the rinse cycle of the wash was the shortest, one would expect the lowest disinfection activity in this case. However, the results show comparable disinfection activity of the HP between the prewash and rinse cycles. Since the cotton carriers of the tested bacteria were added at the beginning of the washing process, the disinfection activity of the HP in the rinse cycle was also partially achieved by the antimicrobial activity of the quaternary ammonium compounds contained in the liquid detergents [34]. In this case, the entire washing process without adding the 3% HP resulted in 1.81, 0.22 and 2.59 log reduction of *E. coli*, *P. aeruginosa* and *S. aureus*, respectively (data not shown). Similar to our study, Hooker et al. [35] demonstrated that adding the HP to the main wash can substantially reduce *E. coli*, *S. aureus* and *P. aeruginosa* (log 6.9) when washing hospital mattresses.

### 2.2. Standard Soil Removal

**Figure 3:**
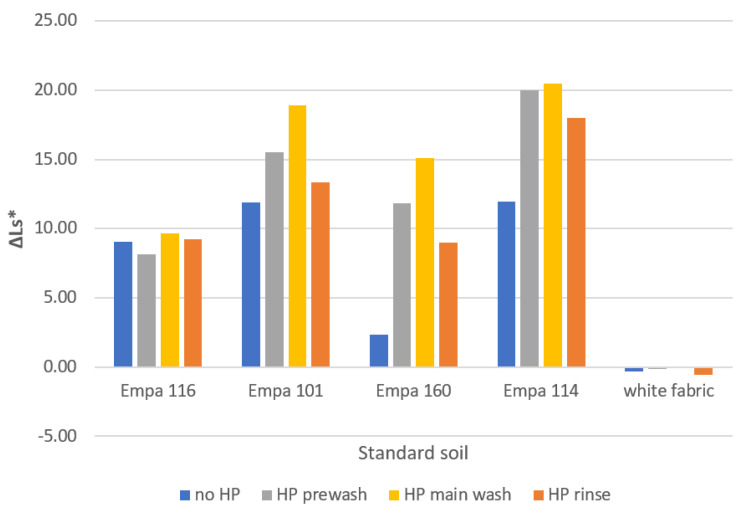
Lightness difference, ΔLs*, of standard soiled fabrics washed at 40 °C with liquid detergent Ox without (no HP) and with the addition of HP in studied wash cycles.

Figure 3 shows the difference in lightness of standard soiled fabrics, ΔLs*, with the addition of 3% HP at different washing cycles. The results show that all standard soils, with the exception of Empa 116 soil (blood/milk/ink), are washed better in the presence of HP. The addition of 3% HP to the main wash is most effective, followed by the prewash and finally the rinse. These results were to be expected, since the oxidation reaction of HP is highly temperature and time-dependent. As mentioned above, both the temperature and time of these wash cycles were lower and shorter than for the main wash. However, unlike the disinfection activity results, where comparable antimicrobial activity was found between the prewash and rinse, this was not the case for soil removal activity.

**Figure 4:**
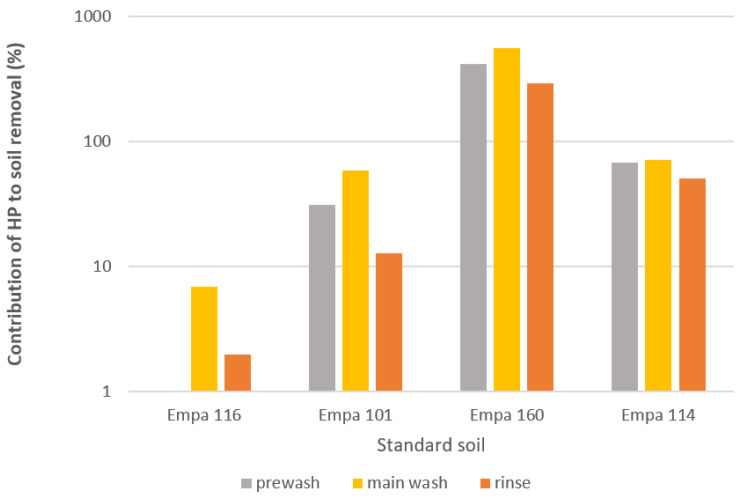
Contribution of HP ^a^ to soil removal after washing in the studied wash cycles. ^a^ Contribution of HP was determined as a ration between ΔLs* of the standard soil sample washed with 3% HP and ΔLs* of the same standard soil sample washed without 3% HP.

**Figure 5:**
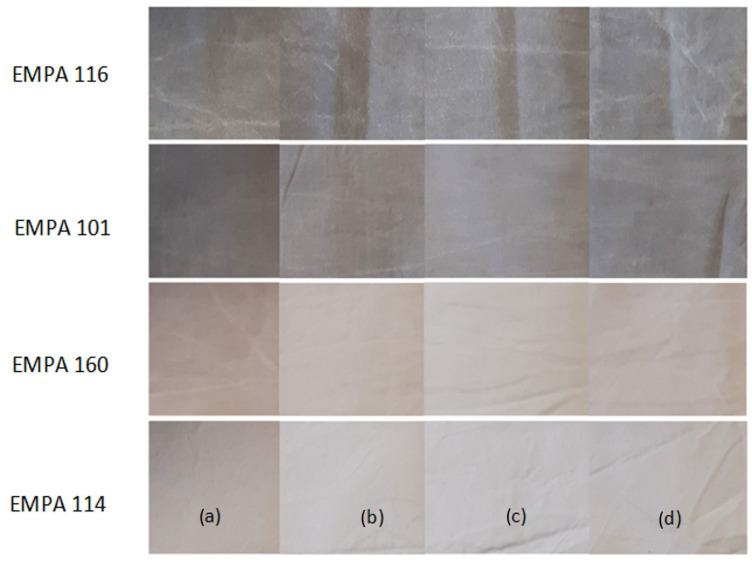
Photos of standard soils after washing without and with the addition of 3% HP in studied wash cycles: (**a**) No HP; (**b**) HP prewash; (**c**) HP main wash; (**d**) HP rinse.

To gain better insight into the soil removal efficiency of 3% HP in each wash, the ratio between the difference in ΔLs* value between the sample of standard soil washed without 3% HP and with it was determined and is shown in Figure 4. Undoubtedly, the addition of 3% HP in the main wash cycle contributed the most to the removal of the studied soils. It was most active against Empa 160 (chocolate cream), followed by Empa 114 (red wine), Empa 101 (carbon black/olive oil) and finally Empa 116 (blood/milk/ink). Thus, the presence of 3% HP in the main wash greatly improved the removal of organic colour pigments into colourless due to its bleaching effect. In the case of standard soil Empa 116, it should be noted that due to the presence of haemoglobin in blood, a reaction occurs between iron and hydrogen peroxide, which leads to oxidation [31,32,36]. As the latter is conducive to disinfection activity, it impairs the formation of bleach ions, which is reflected in lower lightness and poorer soil removal. Accordingly, the addition of 3% HP in the prewash did not contribute to the improved removal of Empa 116 soil, but even slightly affected the washing performance in this wash cycle, as the obtained contribution value was negative. Nevertheless, better removal of this type of soils is obtained at higher pH and temperature [36]. The photos of the studied standard soils without and with the addition of 3% HP in the studied washing cycles are shown in Figure 5.

### 2.3. Colour Change of Textile Samples

**Figure 6:**
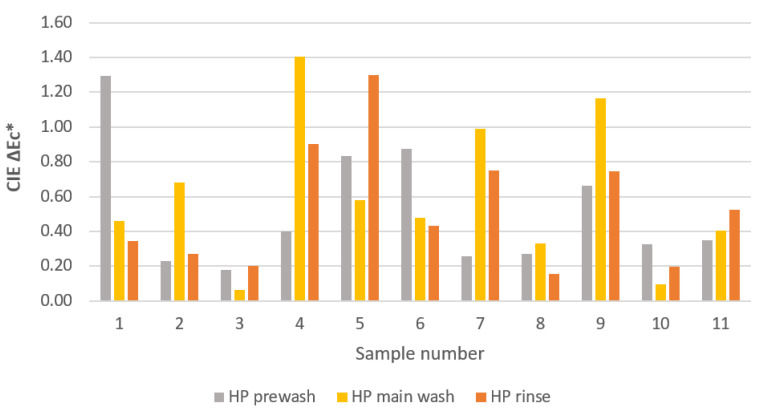
Colour difference, ΔE_c_*, of textile samples determined as the difference of CIE L*a*b* values of the samples after five consecutive washings without and with the addition of 3% HP in the prewash, main wash and rinse cycles.

Figure 6 shows the colour changes of textile samples washed five consecutive times with 3% HP in the studied wash cycles. The results show that 3% HP does not affect the colour of textiles, as the colour difference ΔE_c_* is less than 1.5, which is estimated to be an imperceptible colour difference. The greatest colour changes were expected when hydrogen peroxide was added to the main wash, as the contact time and wash temperature were the longest (30 min) and/or the highest (40 °C). However, this was not the case, as the results show that the colour difference is randomly distributed between the different wash cycles of prewash, main wash and rinse. Accordingly, it can be concluded that the addition of 3% HP to the liquid detergent during a household wash does not cause discoloration in most modern textiles. This can be explained by the fact that modern manufacturers of dyes and auxiliaries have adjusted to the fact that textiles are exposed to various oxidising agents in household laundry. Therefore, the dyes produced and used are resistant to oxidation by hydrogen peroxide [37].

### 2.4. Tensile Strength of Coloured Textile Samples

**Figure 7:**
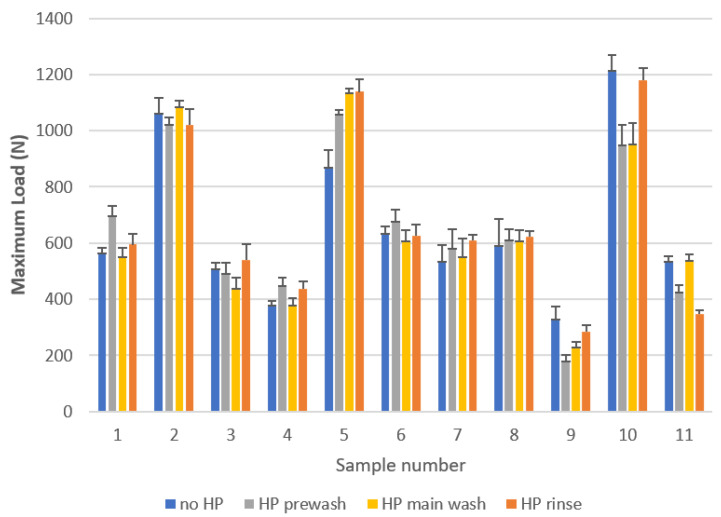
Tensile strength of textile samples after five consecutive washings without and with the addition of 3% HP in the prewash, main wash and rinse cycles at pH 7.

Figure 7 shows the tensile strength of textile samples washed with an addition of 3% HP at different washing cycles. In the statistical analysis, we used a paired *t*-test, bilateral and unilateral, and found that the tensile strength of the samples washed without hydrogen peroxide was not statistically significantly higher than that of the samples washed without peroxide at 95% confidence level. Thus, washing with 3% HP at any cycle of household laundering has no effect on the tensile strength of the washed textiles. At a temperature as low as 40 °C and a relatively short exposure time as used in household washing, hydrogen peroxide does not damage the textile fibres despite its high concentration.

## 3. Materials and Methods

### 3.1. Materials

To test the effectiveness of 3% HP in soil removal, four different EMPA standard soil fabrics were prepared according to the standard SIST EN 60456:2005: Empa 101, Empa 114, Empa 116 and Empa 160, manufactured by Materials Research Products, UK. The properties of the Empa standard soil fabrics used are listed in Table 1.

**Figure 8:**
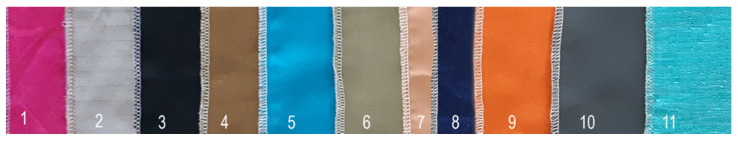
Textile samples of different material composition.

To determine the effect of 3% HP on washed textiles, eleven fabrics (20 × 20 cm) of different material compositions, constructions and colours were prepared. The selected samples are shown in Figure 8, while their composition is shown in Table 2.

### 3.2. Washing Procedure

Washing was performed according to the standard SIST EN 60456:2005. The standard soiled fabrics and the coloured fabric samples were washed separately in a Gorenje household washing machine, model Asko (Gorenje, Slovenia). Household washing was performed at 40 °C using a liquid detergent Ox (Yuco hemija d.o.o., Bački Jarak, Serbia). The ballast load consisted of 100% cotton fabrics (170–190 g/m^2^) with a total weight of 2.45 kg. An appropriate amount of hydrogen peroxide in the form of 35% solution (Belox, Belinka Perkemija d.o.o., Ljubljana-Črnuče, Slovenia) was added to the washing machine in different washing cycles. Separate washing cycles were carried out for determination of disinfection activity, soil removal and colour/tensile strength properties.

The “Universal with prewash” programme was used for all wash cycles. The conditions of the wash cycles are listed in Table 3.

Four different washing procedures were preformed:

1. No HP: washing with liquid detergent only
2. HP prewash: 3% H_2_O_2_ in prewash + wash with liquid detergent
3. HP main wash: washing with liquid detergent + 3% H_2_O_2_ in the main wash
4. HP rinse: washing with liquid detergent + 3% H_2_O_2_ in a rinse wash

For the determination of disinfection efficacy and the determination of soil removal, one washing cycle was performed. For the determination of colour/tensile properties, each procedure was repeated 5 times for each batch of samples. Between each washing procedure, the samples were air dried. The pH of all washing baths was a value of 7.

### 3.3. Analysis

Disinfection efficacy was tested according to the standard EN 16616:2015. In brief: Standard bacterial strains of cultures *E. coli* ATCC 35218, *P. aeruginosa* ATCC 27853 and *S. aureus* ATCC 25923 were taken from the bacterial collection of the Faculty of Health Sciences. The bacteria from the collection were transferred to nutrient agar and incubated at 37 °C for 24 h. Then, a single colony of the strain was transferred from nutrient agar to nutrient broth (Biolife, Milan, Italy) and incubated under the same conditions. For recovery, the bacterial suspension was centrifuged at 3000× *g* for 15 min. The liquid was removed, and the pellet was mixed with an appropriate amount of 0.9% NaCl to obtain a cell concentration of 1.5 × 10^9^ CFU mL^−1^ (OD 620 nm). The suspension was then centrifuged again and resuspended with defibrinated sheep blood in the same volume.

Cotton carriers were cut into 1 cm^2^, boiled in distilled water and autoclaved. The cotton carriers were soaked in the bacterial suspension in defibrinated sheep blood for 15 min at room temperature. After drying at room temperature in a safety cabinet, the carriers were transferred to sterile cotton bags and the disinfection process was carried out in the washing machine. After filling the washing machine with ballast and cotton carriers, 12.5 mL of defibrinated sheep blood per kg of ballast and 5 g/L of liquid detergent were added. Before each wash cycle (i.e., prewash, main wash and rinse), an appropriate amount of 35% HP was added to the washer to achieve a concentration of 3%. At the end of the disinfection step, the bags containing the carriers were removed. Each sample carrier (Ns) was transferred to a separate tube containing a 5 mL neutraliser (0.25 mol/L phosphate buffer) and glass beads. Bacterial cells were detached from the carriers by vortex shaking for 10 min. Serial dilution of the sample liquid was then performed, and 1 mL was transferred to a Petri dish and covered with agar. Endo agar (Merck) was used to obtain *E. coli*, cetrimide agar (Sigma-Aldrich, St. Louis, MO, USA) was used for *P. aeruginosa* and mannitol salt agar (Biolife) was used for *S. aureus*. Agar plates were incubated at 37 °C for 24 h, colonies were counted and results were expressed as log CFU cm^−2^. All experiments were performed with five parallel trials and three replicates. Cotton slides with bacteria not exposed to the washing and disinfection process were considered as control (Nc). The reduction of bacterial cells was expressed as relative reduction (R) in percentage and absolute reduction was calculated as log reduction RL = (Ns/Nc) (log CFU cm^−2^).

To determine the influence of the presence of 3% HP in the studied washing cycles on soil removal and colour change of the tested textile samples, colour measurements were performed using a Datacolour Spectraflesh 600 PLUS-CT spectrophotometer operated with Datacolour Datamaster software with the following settings: Illuminant D65, large area view, specular excluded, UV included and 10° standard observer angle.

For soil removal efficiency determination, the difference in lightness between washed and unwashed standard soils, ΔLs*, was calculated as follows:

(1) $$\Delta L_{S}^{*}=L_{W}^{*}-L_{\mathrm{UN}}^{*}$$

where $L_{W}^{*}$ and $L_{\mathrm{UN}}^{*}$ stands for CIE L* coordinate values of the washed and unwashed standard soils sample.

For colour change determination of the tested coloured textile samples, the colour difference (ΔE*) was calculated using the following equation:

(2) $$\Delta E^{*}=\sqrt{{\left(\Delta L^{*}\right)}^{2}+{\left(\Delta a^{*}\right)}^{2}+{\left(\Delta b^{*}\right)}^{2}}$$

where ΔL*, Δa* and Δb* are differences between the lightness (L*), green-red (a*) and blue-yellow (b*) colour coordinates of the two samples, i.e., samples washed with washing agent in the presence of hydrogen peroxide and samples washed only with washing agent. Five measurements per sample were performed.

Measurements of tenacity at maximum load were performed by an Instron 5567 dynamometer in accordance with SIST ISO 5081:1996. Measurements were performed in the warp direction for ten fabric samples. Prior to testing, the samples were conditioned at 65 ± 2% relative humidity and 20 ± 1 °C for 24 h.

## 4. Conclusions

The introduction of an eco-friendly hydrogen peroxide into household low-temperature (i.e., 40 °C) washing processes can significantly increase the hygiene of the washed textiles and the hygiene of the internal parts of the washing machine by eliminating microorganisms without impairing the intrinsic properties of textiles. Such improved laundry hygiene is of significant importance in preventing the transition of pathogenic microorganisms from person to person through the contact of contaminated textiles during the washing process. Accordingly, the highest disinfection activity against the tested bacteria *E. coli*, *S. aureus* and *P. aeruginosa* was obtained by adding 3% HP to the main wash cycle, corresponding to a reduction > 6.6 log. Despite the relatively high concentration, the addition of 3% HP to the household wash cycle at 40 °C with a liquid detergent does not cause discoloration or damage to textiles, regardless of their raw composition. Moreover, the addition of 3% HP has a positive effect on the removal of standard soiling during different wash cycles, which was highest when added during the main wash. Therefore, it can be concluded that a small addition of eco-friendly HP into the main wash assures disinfection and improved soil removal efficiency of low-temperature washing while maintaining the intrinsic mechanical properties of the textile apparel.

## Figures and Tables

**Table 1:** Code and properties of used Empa standard soiled fabrics.

Code	Description	Mass Per Unit Area (g/m^2^)
Empa 101	100% Cotton soiled with carbon black/olive oil	90
Empa 114	100% Cotton soiled with red wine	200
Empa 116	100% Cotton soiled with blood/milk/ink	200
Empa 160	100% Cotton soiled with chocolate cream	200

**Table 2:** Fabric material compositions.

Sample No.	Component 1	Component 2
1	Cotton	-
2	Cotton	-
3	Viscose	Linen
4	Cotton	Viscose, Linen
5	Cotton	Polyester
6	Viscose	Polyester
7	Cotton	Polyester
8	Wool	Polyester
9	Wool	Viscose, CA
10	Polyester	-
11	Polyester	-

**Table 3:** Washing cycles conditions.

Wash Cycle	Time (min)	Wash Bath Volume (l)	Temperature (°C)
Prewash	16	3	28
Main Wash	30	10	40
Rinse	10	3	26

## Data Availability

Not applicable.

## Sample Availability

Samples are not available from the authors.
